# Medullary Thymic Epithelial Cells and Central Tolerance in Autoimmune Hepatitis Development: Novel Perspective from a New Mouse Model

**DOI:** 10.3390/ijms16011980

**Published:** 2015-01-16

**Authors:** Konstantina Alexandropoulos, Anthony J. Bonito, Erica G. Weinstein, Olivier Herbin

**Affiliations:** Department of Medicine and Clinical Immunology, the Mount Sinai School of Medicine, One Gustave L. Levy Place, Box 1089, New York, NY 10029, USA; E-Mails: anthony.bonito@mssm.edu (A.J.B.); erica.weinstein @mssm.edu (E.G.W.); olivier.herbin@mssm.edu (O.H.)

**Keywords:** autoimmune hepatitis (AIH), T-cells, medullary thymic epithelial cells, medullary thymic epithelial cells (mTECs), central tolerance, peripheral tolerance, autoimmune polyendocrinopathy candidiasis ectodermal dystrophy (APECED), autoimmune polyendocrine syndrome (APS), regulatory T-cells, soluble liver antigen, antinuclear antibodies

## Abstract

Autoimmune hepatitis (AIH) is an immune-mediated disorder that affects the liver parenchyma. Diagnosis usually occurs at the later stages of the disease, complicating efforts towards understanding the causes of disease development. While animal models are useful for studying the etiology of autoimmune disorders, most of the existing animal models of AIH do not recapitulate the chronic course of the human condition. In addition, approaches to mimic AIH-associated liver inflammation have instead led to liver tolerance, consistent with the high tolerogenic capacity of the liver. Recently, we described a new mouse model that exhibited spontaneous and chronic liver inflammation that recapitulated the known histopathological and immunological parameters of AIH. The approach involved liver-extrinsic genetic engineering that interfered with the induction of T-cell tolerance in the thymus, the very process thought to inhibit AIH induction by liver-specific expression of exogenous antigens. The mutation led to depletion of specialized thymic epithelial cells that present self-antigens and eliminate autoreactive T-cells before they exit the thymus. Based on our findings, which are summarized below, we believe that this mouse model represents a relevant experimental tool towards elucidating the cellular and molecular aspects of AIH development and developing novel therapeutic strategies for treating this disease.

## 1. Introduction

Human autoimmune hepatitis (AIH) is a chronic inflammatory disease that targets the liver parenchyma and exhibits a strong female bias. Susceptibility of human patients to AIH has been linked to deviant HLA alleles and compromised antigen presentation [1,2,3,4,5,6,7], and a recent genome-wide association study (GWAS) identified variant HLA alleles that were associated with AIH development [8]. Initiation of the disease is thought to occur through the recognition of liver self-antigens by naive T-lymphocytes [9,10,11]. In addition, impairment of immune regulation is thought to play a critical role in disease development [12,13,14,15,16,17]. AIH is often diagnosed during the late course of the disease, complicating efforts to obtain insight into the immunological events responsible for its initiation. The difficulty in studying autoimmune liver diseases is further complicated by the fact that the liver is an immune-privileged organ. For decades, it has been known that transplanted livers are less frequently rejected than other solid organ transplants. In addition, liver autoimmune diseases, including AIH, develop with a lower frequency compared to other autoimmune conditions, such as type 1 diabetes (T1D) and multiple sclerosis (MS) [18,19,20,21,22]. While animal models are very useful for improving our understanding of the pathophysiology of autoimmune diseases, the tolerogenic properties of the liver have made it difficult to establish a reliable animal model with which to study AIH.

**Figure 1:**
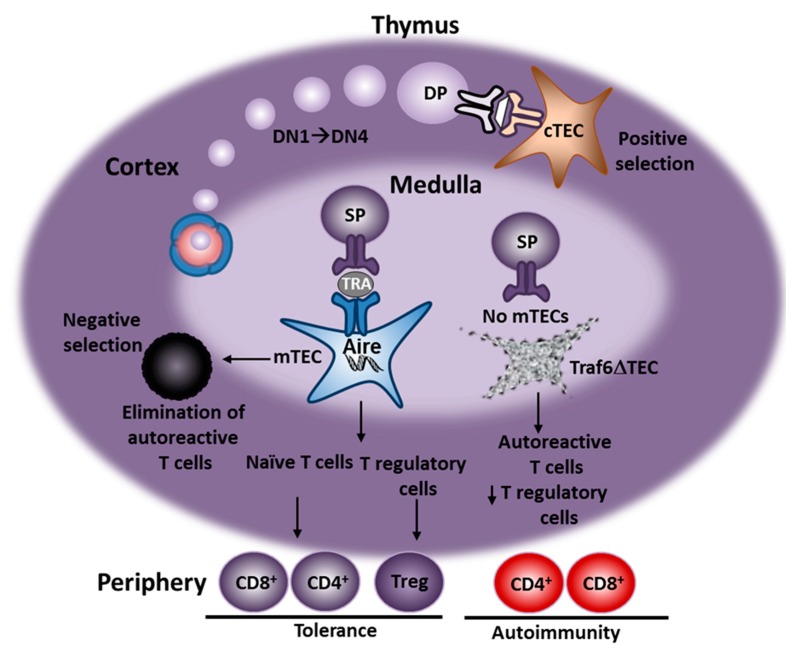
Schematic representation of T-cell selection and tolerance induction in the thymus. Bone marrow progenitors enter the thymus at the corticomedullary junction (CMJ) and migrate towards the cortex, where they differentiate into immature thymocytes lacking expression of the CD4 and CD8 coreceptors (double-negative 1-4, DN1-4). Upregulation of CD4 and CD8 gives rise to CD4^+^CD8^+^ double-positive (DP) thymocytes whose T-cell receptor binds to self-antigens presented by cortical thymic epithelial cells (cTEC). A fraction of DP thymocytes are positively selected and differentiate into CD4^+^ or CD8^+^single-positive (SP) T-cells. These migrate to the medulla, where they bind to tissue-restricted antigens (TRA) presented by medullary TECs (mTEC) and whose expression is regulated by Aire [30,31]. Autoreactive T-cells that bind to TRAs with high affinity are negatively selected. SP T-cells with weak affinity for TRAs are allowed to exit the thymus, whereas T-cells with intermediate affinity for TRAs become regulatory T-cells (Treg). mTEC depletion in Traf6∆TEC mice leads to the production of autoreactive T-cells, impaired production of Tregs and peripheral autoimmunity.

Over the past several years, our research has focused on elucidating the mechanisms that drive T-cell-mediated autoimmunity. Autoimmunity describes the process of an immune response against self, resulting in tissue destruction and, in extreme cases, organ failure. Autoimmune responses involve the activation of the adaptive arm of the immune system with T-lymphocytes playing an especially important role in disease development and progression. T-lymphocytes develop in the thymus from bone marrow precursors, which are subjected to a series of developmental and differentiation steps to become mature T-cells [23,24]. During thymic development, immature T-cells interact with self-antigens expressed by and presented on the surface of thymic stromal cells, namely thymic epithelial cells (TECs) [25]. Based on their localization within the two major compartments of the thymus, the cortex and medulla, TECs are classified as either cortical or medullary TECs (cTECs and mTECs, respectively). cTECs express a variety of self-antigens presented by major histocompatibility complex (MHC) molecules, which determine the diversity of the T-cell repertoire through the process of positive selection. On the other hand, expression of a different set of self-antigens on mTECs generates a self-tolerant T-cell repertoire through the elimination (negative selection) of autoreactive T-cells that bind to medullary self-antigens with high affinity, a process referred to as central tolerance. In addition, self-antigens expressed by mTECs regulate the production of regulatory T-cells (Tregs), which dampen immune responses in peripheral tissues, a process known as peripheral tolerance [25,26,27,28] (Figure 1). Moreover, mTECs serve as a reservoir for unidirectional transfer of self-antigens to other thymic antigen presenting cells (APCs), such as dendritic cells, which, in turn, mediate CD8^+^ T-cell cross-priming [29].

We recently generated conditional knockout mice in which the expression of the ubiquitin ligase, Traf6 (tumor necrosis factor receptor associated-factor 6), was ablated specifically in TECs (Traf6∆TEC mice) [32]. Traf6 deficiency led to dramatically reduced numbers of mTECs, but not cTECs. Despite apparently normal thymocyte differentiation in the thymus, defective mTEC development correlated with spontaneous and chronic liver inflammation, exhibiting pathological features of human AIH, including: perivascular, portal and lobular inflammatory infiltrates; interface hepatitis with infiltration by plasma cells; elevated immunoglobulin levels; and production of anti-nuclear antibodies (ANA) against the liver of Traf6∆TEC animals. A significant increase in liver fibrosis was also detected in knockout animals compared to littermate controls. The spontaneous development of AIH in Traf6∆TEC mice was unexpected and unbiased. As susceptibility to AIH has been linked to specific HLA alleles, suggesting a strong link between MHCII-mediated antigen presentation and disease development [8,33,34], the impaired self-antigen presentation in the thymus of Traf6∆TEC mice may mimic the etiology of human AIH. In addition, AIH development as a result of impaired induction of thymic T-cell tolerance in Traf6∆TEC mice negates the requirement for aggressive, liver-intrinsic perturbations, demonstrating that this model is unique and may recapitulate the mechanisms leading to the development of AIH in human patients. This review briefly summarizes existing animal models of AIH, highlights our findings in the Traf6∆TEC mouse model and discusses its relevance as an appropriate model of AIH.

## 2. Animal Models of AIH

The often late diagnosis of AIH and the difficulty in obtaining samples to study the immune responses involved in disease development necessitate the development of animal models to study this disease. However, the tolerogenic properties of the liver have impeded the establishment of reliable models that recapitulate the chronic/relapsing characteristics of AIH. The high tolerance threshold in the liver is necessary if aberrant immune responses are to be prevented, as the liver is exposed to a variety of different antigens. The detoxifying and drug metabolizing capacity of the liver, exposure to environmental toxins and the steady flow of food and commensal bacterial antigens from the portal circulation necessitate a high threshold of tolerance for the survival of the organ. Several approaches for breaking liver tolerance and generating animal models of AIH have been described over the past several decades. While the different animal models are briefly summarized below, comprehensive reviews of these models have been published elsewhere [22,35,36,37,38].

Early attempts to generate AIH mouse models implicated T-lymphocytes in disease development and provided valuable information on the effector and regulatory mechanisms that govern this process. Initial experiments involved immunization strategies, which consisted of: injections of crude liver homogenates together with activating adjuvants [39]; injections of smooth muscle antibody (SMA) with homogenates of liver-specific proteins and bacterial (*Klebsiella pneumonia*) polysaccharides as adjuvants [40]; and immunization of C57BL/6 mice with a crude 100,000-g supernatant from liver homogenates in complete Freund’s adjuvant (CFA) [41]. Although immunizations induced only transient hepatitis, these experiments nonetheless demonstrated that hepatocyte damage was induced by T-cells.Inbred mouse models injected with syngeneic liver infiltrates were described next without or with neonatal thymectomy [42,43]. These models produced transient hepatitis characterized by perivascular, portal and lobular infiltrates, antibodies to liver-specific proteins and implicated Tregs in the regulation of the autoimmune response. Transgenic mouse models expressing high levels of cytokines typically found in human AIH, such as interferon-γ (IFN-γ) and tumor necrosis factor-α (TNF-α), induced acute liver inflammation, hepatocyte apoptosis and hepatic failure [44,45]. Similarly, systemic administration of concanavalin A (Con A) induced non-specific T-cell activation and resulted in severe liver injury mediated by CD4^+^ T-cells and macrophages [46]. Neutralizing antibodies against TNF-α and IFN-γ inhibited hepatitis development, implicating these cytokines in the Con A-induced inflammatory response against the liver [35,38,47,48].

More recently, genetically engineered mice were generated as part of the effort to establish relevant animal models of AIH. These included transforming growth factor-β (TGF-β) knockout mice that spontaneously developed severe hepatitis and inflammatory lesions in multiple organs, which were associated with lethality within two weeks after birth [49]. Transgenic mouse models were also designed to express self-neoantigens in the liver [22,50,51,52,53,54,55,56]. With the exception of the model by Zierden* et al*. [56], this approach led to tolerance induction, likely due to ectopic expression of the neoantigen in the thymus by mTECs, leading to clonal elimination of neoantigen-reactive T-cells in the thymus before these T-cells were exported to the liver. Tolerance induction following expression of a neoantigen in hepatocytes was shown to be mediated by peripheral deletion of T-cells, anergy and receptor downregulation [57]. These studies largely failed to recapitulate the chronic features of AIH and instead demonstrated the difficulty in breaking liver tolerance against specific antigens. Further limitations of these models included the fact that autoreactive T-cells from a polyclonal repertoire may not behave similarly to transgenic antigens and that these studies mostly dealt with CD8^+^ T-cell responses, whereas AIH susceptibility is linked to MHCII alleles [22,35,36].

As early transgenic mouse models were unable to generate chronic hepatitis and to establish a reliable model of AIH, more recent efforts were focused on the use of viral infections or vaccinations together with expression of human AIH-associated antigens as the means to initiate an immune response against the liver. Vaccination of mice with plasmids expressing a nucleoprotein from the lymphocytic choriomeningitis virus (LCMV) together with interleukin-12 (IL-12) resulted in periportal and lobular infiltrates in the liver, production of viral- and self-antigen reactive T-cells, antibodies against the nucleoprotein and liver injury [58]. A similar model involved vaccination of female mice with cytomegalovirus (CMV) plasmids expressing the antigenic regions of human cytochrome P450 2D6 (CYP2D6) and formiminotransferase cyclodeaminase (FTCD), both proteins targeted by autoantibodies in human type 2 AIH [59,60]. Vaccination of the animals resulted in aminotransferase abnormalities 4–7 months post-immunization, portal and lobular inflammatory infiltrates, liver-infiltrating CD4^+^ and CD8^+^ T-cells, B-cells and antibody production against liver-kidney microsome type 1 (LKM-1) and liver cytosol type 1 (LC1) proteins [38]. The same animal model of type 2 AIH was also used to determine whether central* vs.* peripheral tolerance was responsible for AIH susceptibility in mice. These experiments showed that while low thymic expression of a given liver autoantigen (FTCD) was required, this was not by itself sufficient for AIH development. Rather, decreased peripheral tolerance to the same autoantigen was the main driver of disease development [61].

Infections of animals with viral vectors and human antigens have also been used to break liver self-tolerance by generating a cross-reactive immune response and recruitment of immune cells to the liver. Adenovirus-mediated expression of human CYP2D6 initially produced a strong inflammatory response and liver injury followed by severe hepatitis lasting more than three months [62]. The inflammation was characterized by histological features resembling AIH consisting of hepatic infiltration by CD4^+^ T-cells, antibodies against CYP2D6 and hepatic fibrosis. Another recent mouse model of AIH involved self-limited adenovirus infection with the autoantigen FTCD. This approach led to an initially transient hepatitis followed by chronic AIH that was mediated by CD4^+^ T-cells. The genetic background of the animals (non-obese diabetes, NOD) and viral infection were essential for the development of liver-specific autoimmunity in this experimental setting [63]. While the different models described above recapitulate different aspects of human AIH, the generation of these animal models is liver biased and involves liver-intrinsic perturbations aimed towards overcoming the high tolerance threshold of the liver. These perturbations of immune homeostasis may not be representative of the human condition, and conclusions may be model and antigen dependent [38,64]. In contrast, AIH development in Traf6∆TEC mice was unbiased and occurred spontaneously, in the absence of liver-intrinsic perturbations and as a result of aberrant tolerance induction in the thymus of these mice. Our findings and how this mouse model relates to human AIH are discussed below.

## 3. Generation of Traf6∆TEC Conditional Knockout Mice

To elucidate the mechanisms of T-cell-mediated autoimmunity, we generated an autoimmunity-prone mouse model in which the process of central tolerance,* i.e.*, elimination of autoreactive T-cells in the thymus, was compromised through mTEC depletion [32]. Depletion of mTECs was achieved through conditional ablation of the ubiquitin ligase, Traf6, which regulates mTEC development through activation of the transcription factor NFκB [65]. Floxed Traf6 mice were crossed to “knock-in” mice expressing the Cre recombinase from the FoxN1 promoter [66,67], which regulates the expression of the transcription factor, forkhead box N1 (FoxN1). FoxN1 is an epithelial cell factor specifically expressed in TECs and regulates the development of the thymic organ [68].

Staining of thymic sections with antibodies against medullary markers, such as Aire and the plant lectin, *Ulex europaeus* agglutinin-1 (UEA-1), showed that ablation of Traf6 expression resulted in marked depletion of mTECs, where cTECs were unaffected [32]. These results were confirmed by flow cytometry which also revealed a dramatic reduction in the absolute numbers of mTECs, as a result of Traf6 deletion. Consistent with the previously described role for mTECs in Treg development [69,70,71], the absolute numbers of thymic Tregs were reduced in Traf6**∆**TEC mice. On the other hand, T-cell development, based on the frequency and total numbers of CD4^+^CD8^+^ double-positive (DP) and CD4^+^ and CD8^+^ single-positive (SP) thymocytes, was normal. However, depletion of mTECs was associated with peripheral autoimmune perturbations in Traf6∆TEC mice, presumably due to the generation of an autoreactive T-cell repertoire. The autoimmune symptoms consisted of the presence of autoantibodies, particularly anti-nuclear antibodies (ANAs), against most of the tissues examined. These included the liver, lung, kidney, small and large intestine, adrenal, thyroid and salivary glands, cardiac myocardium and skeletal muscle. In contrast, inflammatory infiltrates (also indicative of peripheral autoimmunity) were mostly confined to the liver and, to a lesser extent, in the lung and kidney of young Traf6**∆**TEC knockout animals, whereas other tissues examined were normal. Despite the presence of autoantibodies and hepatic inflammatory infiltrates, Traf6∆TEC mice lived for at least one year (the longest time point examined); however, the mice were smaller compared to controls and became visibly sick at around six months of age, exhibiting disease symptoms, such as alopecia and inflamed skin, joint swelling and blindness, in addition to liver inflammation (unpublished observations). The clinical score of AIH peaked at around six months and plateaued for the remaining lifespan of the animals. Thus, while mTEC depletion is associated with an inflammatory response against the liver in young Traf6∆TEC mice, impaired mTEC development and function are associated with chronic inflammation affecting several additional tissues in older animals [32].

The importance of mTECs and TRA expression in the development of autoimmunity, including AIH, has been documented in humans expressing mutant forms of Aire. These patients present with autoimmune polyendocrinopathy candidiasis ectodermal dystrophy (APECED) syndrome or APS (autoimmune polyendocrine syndrome) [72,73]. Interestingly, ~20% of humans with APECED/APS syndrome also develop AIH [74,75], characterized by autoantibodies against cytochrome P450 2A6 and 1A2 [76,77,78]. Likewise, Aire-deficient mice develop serum autoantibodies and inflammatory infiltrates in different tissues, including the liver [79]. The hepatic inflammation associated with Aire deficiency in mice was recently shown to recapitulate various aspects of APS type 1 (APS-1)-associated AIH, suggesting that impaired central tolerance is an important factor in AIH development. Based on these observations and given the defects in central tolerance in Traf6∆TEC mice, we believe that these animals represent a relevant model with which to study the pathogenic mechanisms of AIH and to explore new therapeutic approaches to better manage the disease.

## 4. Characterization of AIH in Traf6∆TEC Mice

AIH is a complex disease whose etiology is unknown. It may involve one or more genes operating alone or together, and it may be triggered by environmental factors in genetically predisposed individuals. The diagnosis is based on histological abnormalities that include, but are not limited to: the presence of portal and lobular inflammation and interface hepatitis; biochemical markers, such as increased levels of alanine aminotransferase (ALT); abnormal levels of serum immunoglobulins that include autoantibodies; and lymphocytic infiltrates consisting of T- and B-cells [1,10,11,80,81,82,83]. While the spectrum of inflammatory infiltrates in Traf6∆TEC mice was limited, 100% of the animals developed liver inflammation compared to 50% penetrance in the lung and 30% penetrance in the kidney of knockout mice. Because, as mentioned above, the liver has a high tolerogenic capacity, the high penetrance of the inflammation in the liver of Traf6∆TEC animals prompted us to take a closer look. Histologically, the inflammation in the liver was detected as early as four weeks after birth (the earliest time point tested), and it was chronic, as histopathological scores peaked at six months after birth and persisted throughout the lifetime of the animals. The hepatic inflammation in knockout mice recapitulated all known histopathological and immunological aspects of human AIH, including portal and lobular infiltrates and interface hepatitis with spreading of the infiltrates into the liver parenchyma. Biochemically and similar to human AIH, the infiltration was accompanied by significantly increased ALT levels with a mean value of ~100 U/L and increased periportal fibrosis [32]. There were no gender-specific differences in the penetrance of AIH in knockout mice, as 100% of all mice exhibited liver inflammation.

Autoantibodies are the serological hallmarks of AIH, and their presence in patients with hepatitis is used for diagnostic purposes [81]. Two different categories of AIH have been described: type 1, which is characterized by the presence of ANAs and anti-smooth-muscle (SMA) antibodies; and type 2, which is associated with the presence of anti-LKM-1 or -LC-1 antibodies [9,84,85]. Approximately 65% of AIH patients present with ANAs or anti-SMA, whereas 58% of patients with type 1 AIH generate antibodies against soluble liver antigen (SLA) in the presence or absence of ANAs or anti-SMA [11]. Anti-SLA antibodies display a greater specificity for AIH than ANAs or anti-SMA antibodies and may be useful in AIH diagnosis when conventional autoantibodies are not present in the sera of patients [86]. Autoantibodies against the cytochrome P450 enzyme (CYP) system, specifically CYP1A2 and CYP2A6, have also been found in patients with APECED-related AIH [74,78]. Interestingly, we found that sera from the great majority of Traf6∆TEC mice had significantly increased levels of total, as well as specific (IgG1, IgM) immunoglobulins. Anti-nuclear and anti-SLA antibodies were significantly increased, whereas in contrast, anti-SMA and -LMK-1 antibody levels were similar to control mice, suggesting that the liver inflammation in Traf6∆TEC mice mimics aspects of human type 1 AIH. Whether the Traf6∆TEC mice also generate serum autoantibodies against the mouse equivalents of CYP1A2 or CYP2A6 (given that, similar to APECED patients central tolerance is compromised in these mice), was not examined in these experiments.

Given the increased production of immunoglobulins and autoantibodies, we also examined if plasma cells were present in the hepatic inflammatory lesions of Traf6∆TEC mice. Staining of liver sections with methyl green pyronin revealed the significantly increased presence of plasma cells within portal inflammatory lesions. In addition, analysis of hepatic hematopoietic cell suspensions with antibodies against plasma cell markers (CD138 and CD19) also showed increased numbers of plasma cells in the liver of knockout animals [32]. Together, these results suggest that the liver inflammation in Traf6∆TEC mice recapitulates several hallmarks of human AIH, including the chronic nature and histopathological features of the disease, as well as immunological parameters, such as the presence of plasma cells and the production of autoantibodies relevant to type 1 AIH.

Consistent with the idea that the Traf6∆TEC mice represent a mouse model relevant to human AIH, our results were similar to those obtained with a mouse model of human APS-1-associated AIH that was recently described by Hardtke-Wolenski* et al.* [87]. The approach involved the generation of Aire-deficient mice through deletion of exon 2 of the *aire* gene (Aire-∆ex2) in the Balb/c genetic background. Most Aire-∆ex2 mice presented with multi-organ autoimmunity, while 24% of these animals developed AIH with a disease penetrance comparable to that of human APS-1. Similar to our Traf6∆TEC mice, the inflammation was characterized by periportal inflammatory infiltrates and elevated aminotransferase levels indicative of immune-mediated liver damage.

The development of AIH in both mouse models was spontaneous and chronic and presumably due to impaired antigen presentation and central tolerance in the thymus. As we found in the Traf6∆TEC mice, Aire deletion resulted in a break of humoral tolerance with a preponderance of B-cells in the liver in Aire-∆ex2 mice and the generation of serum autoantibodies against liver proteins. The humoral responses of Traf6∆TEC and Aire-∆ex2 mice were not directed against a single or a few dominant autoantigens [32,87]. This was in contrast to other organ-specific autoimmune diseases, such as multiple sclerosis and type 1 diabetes. Despite the similarities, there were also differences between the Traf6∆TEC and Aire-∆ex2 mouse models in terms of the penetrance of AIH (100% *vs.* 24%, respectively) and the transaminases involved. In addition, while the Traf6∆TEC mice developed mostly antinuclear, Aire-∆ex2 mice displayed mostly cytoplasmic autoantibodies. The differences between the two models are likely due to the effect of the mutations on self-antigen expression in the thymus. For example, while Aire deficiency would affect the expression of Aire-dependent antigens, mTEC depletion would have a more global effect on the expression of both Aire-dependent, as well as Aire-independent self-antigens. This is supported by the findings of Hardtke-Wolenski* et al.*, in that different Aire mutations differentially affected AIH development in Aire-∆ex2 mice [87]. Despite the differences, the results obtained from the two mouse models are in line with the humoral phenotype of human type 1 AIH, which appears to be polyclonal and not directed against a single dominant autoantigen. A comparison of the immunological features of AIH in Traf6∆TEC and Aire-∆ex2 mice is shown in Table 1.

**Table 1:** Comparison of the phenotypic features of Traf6∆TEC and Aire-∆ex2 mouse models.

Mouse Model	Traf6∆TEC	Aire-∆ex2
Parameters		
Genetic Background	C57BL/6	Balb/c
Thymus		
Central tolerance	Impaired	Impaired
mTEC number	Depleted	Normal *
Treg production	Reduced (50%)	Normal *
Periphery		
AIH		
Features	Chronic/Spontaneous	Chronic/Spontaneous
Penetrance	100%	24%
Aminotransferases	ALT	AST
Portal infiltrates	CD4^+^ T cells, CD19^+^CD138^+^ Plasma cells	CD4^+^ T cells, B cells
Autoantibodies	Mostly ANA, anti-SLA, Polyclonal	Mostly cytoplasmic, Polyclonal
Intrahepatic autoantigens	Present	Present
Cytokines		
Intrahepatic	Increased (IFN-γ, IL-4, IL-10, TGF-β)	ND
Serum	Normal	Increased (TNF-α, IL-2, IL-9)
Tregs		
Hepatic	Increased	Increased
Splenic	Normal	Normal

* Although mTEC and Treg numbers were not directly analyzed in the Aire-∆ex2 mice, adult Aire-deficient mice exhibit normal numbers of mTECs and thymic Tregs [79,88]. ALT = alanine aminotransferase; AST = aspartate aminotransferase; ANA = antinuclear antibodies; SLA = soluble liver antigen; ND = not determined.

## 5. AIH in Traf6∆TEC Mice Is T-Cell-Dependent

Early studies on the pathogenesis of AIH showed that within areas of interface hepatitis, T-lymphocytes predominated over other cell types. Within the T-lymphocyte population, CD4^+^ T-cells were the dominant T-cell subset, with CD8^+^ T-cells making up a minor population in human [9,82,83,89] and murine AIH [43,63,87]. Liver inflammation and associated liver damage in AIH is thought to be mediated by CD4^+^ T-cells that aberrantly recognize self-antigens presented by HLA alleles on the surface of professional APCs [1,9,80]. This idea is consistent with genetic evidence and GWAS in humans linking the expression of certain HLA molecules and antigen presentation by MHCII alleles with susceptibility to AIH [2,8,34,90]. Interestingly, similar results were observed in Traf6∆TEC mice in that the portal inflammation consisted primarily of CD4^+^ T-cells with fewer CD8^+^ T-cells present in the lesions, whereas CD8^+^ T-cells were enriched in the liver parenchyma (unpublished observations) [32]. Consistent with these findings, there were increased absolute numbers of both CD4^+^ and CD8^+^ T-cells in the liver of knockout animals compared to controls. The accumulation of T-cells was specific to the liver, as analysis of multiple animals revealed no significant differences in the numbers of T-cells in other tissues, including the lung and kidney of wild-type* vs.* Traf6∆TEC mice. These results were consistent with the histological analysis of tissues from knockout animals showing predominant liver inflammation in these mice. In addition to the increased numbers of CD4^+^ and CD8^+^ T-cells, a third population was present in the liver of Traf6∆TEC mice, which expressed both the CD4^+^ and CD8^+^ co-receptors. CD4^+^CD8^+^ T-cells have been found in the peripheral blood of patients with different autoimmune diseases [91,92]. However, the function of these cells and whether they contribute to disease development is presently unknown. Similarly, it is not clear whether these cells also exist in the peripheral blood of AIH patients and, if so, whether they contribute to disease development and/or maintenance. While in Traf6∆TEC mice, the CD4^+^CD8^+^ cells were found in large enough numbers to purify for* in vitro* analyses or adoptive transfers into immune-deficient recipients, wild-type mice exhibited very low numbers of these cells, a fact that limited studies to elucidate their function [32]. Nonetheless, it may be of interest to examine whether these cells are also present in the peripheral blood of AIH patients and whether their presence is associated with AIH development.

Analysis of the phenotype and properties of T-cells from control and Traf6∆TEC mice revealed the presence of activated CD4^+^ and CD8^+^ T-cells in the liver of knockout animals [32]. The use of mixed lymphocyte reactions (MLRs), in which syngeneic and allogeneic APCs were used to stimulate CD4^+^ T-cells* in vitro*, revealed that the liver of Traf6∆TEC animals contained autoreactive T-cells responding to liver-specific antigens. In these experiments, liver APCs were incubated with liver T-cells, and T-cell activation was measured by T-cell proliferation* in vitro*. Syngeneic APCs isolated from the liver of knockout animals potently stimulated liver T-cells from the same animals compared to unstimulated controls or wild-type T-cells stimulated by wild-type syngeneic APCs. Similar experiments further showed that Traf6∆TEC liver T-cells only responded to liver, but not to lung or kidney, APCs presenting endogenous, tissue-specific antigens. Furthermore, knockout liver T-cells were stimulated by* in vitro* differentiated DCs loaded with liver lysates, but not by DCs loaded with lung or kidney extracts, suggesting that the liver of Traf6∆TEC animals contains autoreactive T-cells that respond to liver autoantigens. The presence of autoreactive T-cells was specific to the liver, as splenic T-cells from wild-type or Traf6∆TEC mice responded similarly to allogeneic or syngeneic stimulation. These experiments also revealed that allogeneic APC stimulation of liver T-cells was much less robust compared to allogeneic stimulation of their splenic counterparts, consistent with the high tolerance threshold of the liver. The increased presence of autoreactive T-cells in the liver of Traf6∆TEC animals correlated with increased intrahepatic production of the Th1 and Th2 cytokines, IFN-γ and interleukin-4 (IL-4), respectively. In contrast, splenic T-cell activation and cytokine presence in the sera of knockout animals were similar to controls, indicative of normal, systemic T-cell responses in Traf6∆TEC mice.

Similar results were observed in the Aire-∆ex2 mice, which exhibited increased intrahepatic presence and proliferation of CD4^+^ T-cells. Whether CD4^+^CD8^+^ T-cells were also present in the liver of these mice was not reported. The authors suggested that the increased intrahepatic presence and proliferation of CD4^+^ T-cells was indicative of an active intrahepatic inflammatory response likely driven by hepatic autoantigens, as was the case in Traf6∆TEC mice. Consistent with this and our findings in Traf6∆TEC mice, the humoral and cellular immune responses in Aire-∆ex2 mice were confined to the liver and were not observed in the spleen of the same animals. While the inflammation in the liver of Aire-∆ex2 mice was accompanied by increased serum cytokine levels (in contrast to the Traf6∆TEC mice, which exhibited normal serum cytokines), these did not correlate with histological activity (Table 1) [87]. Together, these results suggest that defects in central tolerance in the Traf6∆TEC and Aire-∆ex2 mouse models correlate with liver-specific inflammatory responses against liver autoantigens, a mechanism that may be in effect in APS-1-associated AIH.

As previously reported [43,63] and to further examine whether the aberrant T-cell responses observed in the liver of Traf6∆TEC mice were responsible for AIH development, liver T-cells were adoptively transferred into Rag1-deficient recipients, which lack T- and B-cells. Total CD3^+^ T-cell transfer led to AIH development of increased severity (based on clinical scores and ALT levels) compared to the donor mice within 4–6 weeks after transfer. There was widespread portal inflammation and interface hepatitis in the liver of Traf6∆TEC T-cell recipients, which associated with increased fibrosis and ALT levels compared to donor mice and Rag1^−/−^ recipients of wild-type liver T-cells. In contrast, transfer of sorted liver CD4^+^ T-cells or serum revealed no significant differences in the clinical scores or ALT levels between Rag1^−/−^ recipients of wild-type or Traf6∆TEC liver T-cells, although some infiltrates were present in recipients of knockout cells. Likewise, transfer of either control or Traf6∆TEC CD8^+^ T-cells had no effect on AIH histopathological scores or ALT levels of recipient mice, suggesting that both CD4^+^ and CD8^+^ T-cells are required for AIH development in Traf6∆TEC mice [32].

**Figure 2:**
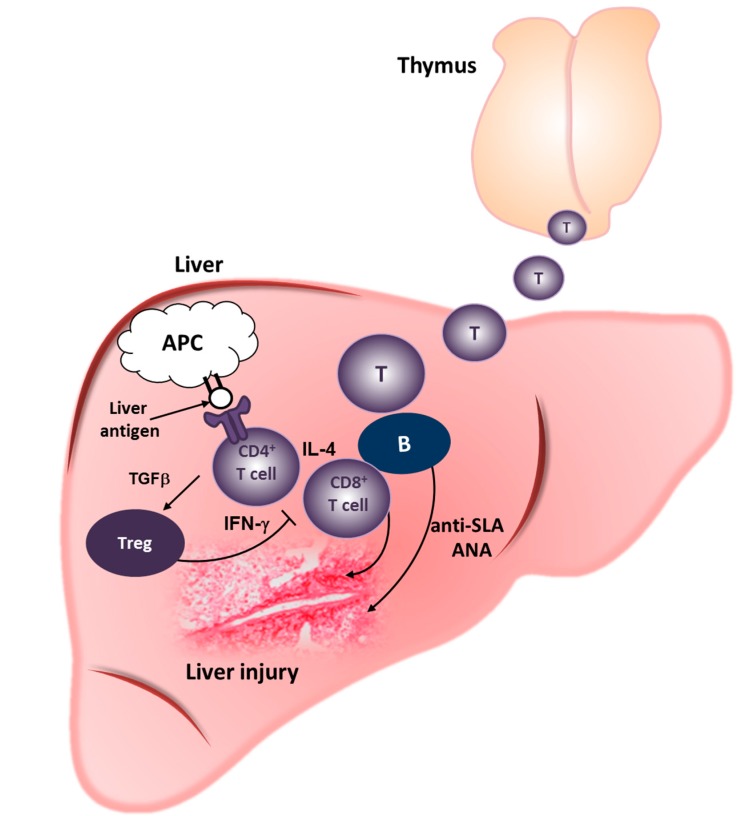
Working model of AIH induction in Traf6∆TEC mice. mTEC depletion in the thymus of Traf6∆TEC mice results in the production of autoreactive CD4^+^ T-cells, which home to the liver. In the liver, autoreactive CD4^+^ T-cells aberrantly recognize liver-specific antigens presented by APCs, leading to T-cell activation. Activated T-cells differentiate into Th1 or Th2 effector cells, secreting IFN-γ or IL-4, respectively. IFN-γ mediates the activation of cytotoxic CD8^+^ T-cells, whereas IL-4 induces the formation of plasma cells and autoantibody production, including ANAs and anti-SLA, leading to hepatocyte destruction and liver injury. Liver antigen-specific Tregs produced locally in response to inflammation counteract effector T-cell-mediated inflammation, leading to chronic AIH.

Consistent with our results that systemic responses (splenic T-cell activation, proliferation and cytokine production) in Traf6∆TEC mice were normal, transfer of CD3^+^ splenic T-cells from Traf6∆TEC mice into Rag1^−/−^ recipients had no effect on the liver. Therefore, antigen recognition and development of inflammation in Traf6∆TEC mice is liver intrinsic with no involvement of systemic responses. These observations are supported by previous evidence that antigen presentation to both CD4 and CD8 effector T-cells can occur intrahepatically, without the need for trafficking of T-cells to peripheral lymphoid organs [93,94,95]. Together, these findings suggest that production in the thymus and homing of CD4^+^autoreactive T-cells to the liver in Traf6∆TEC mice results in the formation of portal infiltrates. Recognition of liver antigen(s) by autoreactive CD4^+^ T-cells initiates an inflammatory response leading to chronic inflammation and liver injury. Our working model of AIH development in Traf6∆TEC mice is shown and discussed in Figure 2.

Our findings are consistent with previously proposed models of AIH development and provide experimental evidence for the hypothesis that the development of AIH is due to faulty antigen recognition by aberrantly-activated T-lymphocytes [1,9,11,80]. While a role for NK cells and macrophages in the development of AIH has been proposed, the involvement of these cells in disease development in our mouse model has not as yet been explored. In addition, while Th17 cells have been implicated in autoimmunity development, whether Th17 cells play a role in AIH development is currently unknown. Th17 cells have been shown to be increased in the peripheral blood and liver of AIH patients [96]; however, the role of these cells in disease development is still unclear. In animal models of AIH, the existing evidence is conflicting concerning the role of IL-17 in AIH development. While Yan* et al.* recently showed that IL-17 is critical for the induction of AIH in the Con A animal model of AIH [97], others have shown that IL-17 is less important than IFN-γ or not important at all in disease development [98,99,100]. In the Traf6∆TEC mice, we found no difference in IL-17 levels, either in the liver or the serum (unpublished observations) of knockout mice compared to controls, suggesting that Th17 cells are unlikely to play a role in AIH development in this animal model.

## 6. Role of Tregs in AIH Development in Traf6∆TEC Mice

As mentioned above, mTECs regulate the production of Tregs in the thymus, and mTEC depletion in Traf6∆TEC mice led to a 50% reduction in the thymic output of Tregs [32]. Despite this reduction, there was a marked increase in Treg numbers in the liver of knockout mice compared to controls, which was associated with the increased production of the immunosuppressive cytokines, TGF-β and IL-10. Incubation of Traf6∆TEC liver CD4^+^CD25^−^ T-cells* in vitro* in the presence of TGF-β resulted in the increased frequency of CD25^+^Foxp3^+^ T-cells in the cultures compared to controls, suggesting that Foxp3^+^ Tregs may be produced in the liver of knockout animals in response to the inflammatory environment induced by autoreactive T-cells. The role liver Tregs play in AIH pathogenesis is not altogether clear. Early studies associated AIH development with defective immune regulation [12,13,14]. Subsequent studies have shown that Tregs from pediatric type 2 AIH patients display impaired capacity to control the proliferation of CD4^+^ and CD8^+^ effector T-cells, and reduced numbers of Tregs have been found in AIH patients [15,16,17,93]. However, recent reports found no impairment in the numbers of Tregs in the peripheral blood of AIH patients. Instead, the intrahepatic frequency of these cells was higher and correlated with the degree of the inflammation in the liver of these patients [82,83,101]. It was argued that the discrepancies may be due to immunotherapy-induced reductions in Treg numbers and changes in Treg phenotypic markers as a result of disease activity and/or treatment [101,102]. The increased intrahepatic frequency of Tregs in AIH patients reported by Peiseler* et al* [82] and Taubert* et al.* [83] is in agreement with our data showing increased numbers of Tregs in the liver of Traf6∆TEC mice. In addition, Hardtke-Wolenski* et al.* also found increased Treg numbers and proliferation in the liver of Aire-∆ex2 mice, whereas splenic Tregs were normal (Table 1) [87]. Therefore, intrahepatic accumulation of Tregs may reflect a similar mechanism of AIH regulation in the Traf6∆TEC and Aire-∆ex2 mouse models and humans with AIH.

It is currently unclear whether the Tregs in the liver of Traf6∆TEC animals are functional, as the assessment of the suppressive function of these cells* in vitro* was limited by the numbers of Tregs that could be purified from wild-type mice. In the case of the spleen, despite the reduction in Treg numbers produced in the thymus, the numbers and suppressive function of splenic Tregs in Traf6∆TEC mice were normal. However, we cannot extrapolate that liver Tregs also exhibit normal suppressive function, as peripheral T-cell responses were normal in knockout animals. Therefore, it is possible that despite the increased production of Tregs in the liver of Traf6∆TEC mice, these cells are incapable of suppressing the inflammatory response initiated by autoreactive T-cells leading to AIH development and liver injury. On the other hand, given the increased production of hepatic TGF-β and IL-10 in knockout animals and evidence suggesting that antigen-specific Tregs normally limit liver inflammation, it is likely that the Tregs produced in the liver of Traf6∆TEC mice are indeed functional. Alternatively, the hepatic inflammation in Traf6∆TEC mice may be due to the reduced susceptibility of effector T-cells to Treg-mediated suppression, as CD4^+^ T-cells isolated from AIH patients were shown to exhibit reduced expression of the inhibitory receptor, Tim-3, which binds to galectin-9 on Tregs and induces effector T-cell death [10,103].

Functional Foxp3^+^ Tregs in the liver of knockout animals may be produced in response to inflammation, so that tolerance and inflammation are mutually limiting, leading to an equilibrium that contributes to the chronic nature of AIH (Figure 2). Such regulation of inflammation/tolerance induction can be mediated by liver APCs, particularly liver sinusoidal endothelial cells (LSECs), which regulate T-effector responses or the induction of tolerance. LSECs express high levels of MHCII and costimulatory molecules and can capture and process soluble antigen to activate CD4^+^ T-cells [104,105]. Priming of CD4^+^ T-cells by LSECs results in Foxp3 expression and differentiation of CD4^+^ T-cells into Tregs, leading to the suppression of AIH [36,101,106,107]. Whether these cells also regulate the production of Foxp3^+^ T-cells in the liver of Traf6∆TEC mice awaits further experimentation. Further experiments are also needed to elucidate the interplay of effector T-cells to Tregs in the liver of Traf6∆TEC mice and whether the mechanisms that regulate inflammation* vs.* tolerance in Traf6∆TEC mice are similar to those of human AIH.

## 7. Conclusions

We have described a novel mouse model of autoimmune hepatitis that, to a great extent, recapitulates the histopathological and immunological characteristics of the human condition. The generation of the model involved liver-extrinsic perturbations, which unexpectedly resulted in the loss of liver tolerance. We believe that this mouse model is physiologically relevant to human AIH based on genetic evidence that faulty antigen presentation may be responsible for disease development. In addition, the similarity of the Traf6∆TEC to Aire-∆ex2 mice, which were developed as a model for human APS-1-associated AIH, also supports the relevance of the Traf6∆TEC mouse model to human AIH. However, further work is needed to establish that this animal model can be used as a preclinical tool to study the etiology of AIH. For example, the identity of the liver antigen(s) targeted by autoreactive T-cells in Traf6∆TEC mice is still unclear. However, the observation that these mice specifically produce ANAs and anti-SLA antibodies suggests that similar antigens may be targeted by autoreactive T-cells in Traf6∆TEC mice and humans with type 1 AIH. Further studies with “humanized” mice, such as transgenic animals expressing human HLA alleles in the Traf6∆TEC background, will be needed to determine whether endogenous mouse antigens can be recognized by mouse T-cells in the context of human MHCII alleles and to determine the nature of the pathogenic peptides/proteins involved in AIH development. In addition, because we found that antibodies in the sera of Traf6∆TEC mice recognize novel proteins in liver extracts compared to sera of control animals [32], identification of these proteins and their human counterparts may lead to the characterization of new targets and the development of novel therapeutic approaches to manage or treat AIH.

## References

[1] Liberal R., Longhi M.S., Mieli-Vergani G., Vergani D. (2011). Pathogenesis of autoimmune hepatitis. Best Pract. Res. Clin. Gastroenterol..

[2] Donaldson P.T. (2002). Genetics in autoimmune hepatitis. Semin. Liver Dis..

[3] Donaldson P.T. (2004). Genetics of liver disease: Immunogenetics and disease pathogenesis. Gut.

[4] Vergani D., Choudhuri K., Bogdanos D.P., Mieli-Vergani G. (2002). Pathogenesis of autoimmune hepatitis. Clin. Liver Dis..

[5] Czaja A.J., Carpenter H.A., Santrach P.J., Moore S.B. (1993). Significance of HLA DR4 in type 1 autoimmune hepatitis. Gastroenterology.

[6] Czaja A.J., Carpenter H.A., Santrach P.J., Moore S.B. (1993). Genetic predispositions for the immunological features of chronic active hepatitis. Hepatology.

[7] Oliveira L.C., Porta G., Marin M.L., Bittencourt P.L., Kalil J., Goldberg A.C. (2011). Autoimmune hepatitis, HLA and extended haplotypes. Autoimmun. Rev..

[8] De Boer J., Williams A., Skavdis G., Harker N., Coles M., Tolaini M., Norton T., Williams K., Roderick K., Potocnik A.J. (2003). Transgenic mice with hematopoietic and lymphoid specific expression of Cre. Eur. J. Immunol..

[9] Mieli-Vergani G., Vergani D. (2011). Autoimmune hepatitis. Nat. Rev. Gastroenterol. Hepatol..

[10] Liberal R., Grant C.R., Mieli-Vergani G., Vergani D. (2013). Autoimmune hepatitis: A comprehensive review. J. Autoimmun..

[11] Heneghan M.A., Yeoman A.D., Verma S., Smith A.D., Longhi M.S. (2013). Autoimmune hepatitis. Lancet.

[12] Hodgson H.J., Wands J.R., Isselbacher K.J. (1978). Alteration in suppressor cell activity in chronic active hepatitis. Proc. Natl. Acad. Sci. USA.

[13] Nouri-Aria K.T., Hegarty J.E., Alexander G.J., Eddleston A.L., Williams R. (1982). Effect of corticosteroids on suppressor-cell activity in “autoimmune” and viral chronic active hepatitis. N. Engl. J. Med..

[14] Vento S., Hegarty J.E., Bottazzo G., Macchia E., Williams R., Eddleston A.L. (1984). Antigen specific suppressor cell function in autoimmune chronic active hepatitis. Lancet.

[15] Ferri S., Longhi M.S., de Molo C., Lalanne C., Muratori P., Granito A., Hussain M.J., Ma Y., Lenzi M., Mieli-Vergani G. (2010). A multifaceted imbalance of T cells with regulatory function characterizes type 1 autoimmune hepatitis. Hepatology.

[16] Longhi M.S., Ma Y., Bogdanos D.P., Cheeseman P., Mieli-Vergani G., Vergani D. (2004). Impairment of CD4^+^CD25^+^ regulatory T-cells in autoimmune liver disease. J. Hepatol..

[17] Longhi M.S., Ma Y., Mitry R.R., Bogdanos D.P., Heneghan M., Cheeseman P., Mieli-Vergani G., Vergani D. (2005). Effect of CD4^+^CD25^+^ regulatory T-cells on CD8 T-cell function in patients with autoimmune hepatitis. J. Autoimmun..

[18] Calne R.Y., Sells R.A., Pena J.R., Davis D.R., Millard P.R., Herbertson B.M., Binns R.M., Davies D.A. (1969). Induction of immunological tolerance by porcine liver allografts. Nature.

[19] Qian S., Demetris A.J., Murase N., Rao A.S., Fung J.J., Starzl T.E. (1994). Murine liver allograft transplantation: Tolerance and donor cell chimerism. Hepatology.

[20] Kamada N. (1985). The immunology of experimental liver transplantation in the rat. Immunology.

[21] Crispe I.N. (2003). Hepatic T cells and liver tolerance. Nat. Rev. Immunol..

[22] Hardtke-Wolenski M., Jaeckel E. (2010). Mouse models for experimental autoimmune hepatitis: Limits and chances. Dig. Dis..

[23] Zlotoff D.A., Bhandoola A. (2011). Hematopoietic progenitor migration to the adult thymus. Ann. N. Y. Acad. Sci..

[24] Yang Q., Jeremiah Bell J., Bhandoola A. (2010). T-cell lineage determination. Immunol. Rev..

[25] Alexandropoulos K., Danzl N.M. (2012). Thymic epithelial cells: Antigen presenting cells that regulate T cell repertoire and tolerance development. Immunol. Res..

[26] Klein L., Kyewski B., Allen P.M., Hogquist K.A. (2014). Positive and negative selection of the T cell repertoire: What thymocytes see (and don’t see). Nat. Rev. Immunol..

[27] Klein L., Hinterberger M., Wirnsberger G., Kyewski B. (2009). Antigen presentation in the thymus for positive selection and central tolerance induction. Nat. Rev. Immunol..

[28] Mouchess M.L., Anderson M. (2014). Central tolerance induction. Curr. Top. Microbiol. Immunol..

[29] Perry J.S., Lio C.W., Kau A.L., Nutsch K., Yang Z., Gordon J.I., Murphy K.M., Hsieh C.S. (2014). Distinct contributions of Aire and antigen-presenting-cell subsets to the generation of self-tolerance in the thymus. Immunity.

[30] Laan M., Peterson P. (2013). The many faces of aire in central tolerance. Front. Immunol..

[31] Peterson P., Org T., Rebane A. (2008). Transcriptional regulation by AIRE: molecular mechanisms of central tolerance. Nat. Rev. Immunol..

[32] Bonito A.J., Aloman C., Fiel M.I., Danzl N.M., Cha S., Weinstein E.G., Jeong S., Choi Y., Walsh M.C., Alexandropoulos K. (2013). Medullary thymic epithelial cell depletion leads to autoimmune hepatitis. J. Clin. Investig..

[33] Donaldson P.T., Doherty D.G., Hayllar K.M., McFarlane I.G., Johnson P.J., Williams R. (1991). Susceptibility to autoimmune chronic active hepatitis: Human leukocyte antigens DR4 and A1-B8-DR3 are independent risk factors. Hepatology.

[34] Strettell M.D., Donaldson P.T., Thomson L.J., Santrach P.J., Moore S.B., Czaja A.J., Williams R. (1997). Allelic basis for HLA-encoded susceptibility to type 1 autoimmune hepatitis. Gastroenterology.

[35] Christen U., Hintermann E., Jaeckel E. (2009). New animal models for autoimmune hepatitis. Semin. Liver Dis..

[36] Jaeckel E., Hardtke-Wolenski M., Fischer K. (2011). The benefit of animal models for autoimmune hepatitis. Best Pract. Res. Clin. Gastroenterol..

[37] Hardtke-Wolenski M., Noyan F., Jaeckel E. (2011). Requirements and challenges of a preclinical autoimmune hepatitis mouse model. Dig. Dis..

[38] Czaja A.J. (2010). Animal models of autoimmune hepatitis. Expert Rev. Gastroenterol. Hepatol..

[39] Scheiffarth F., Warnatz H., Mayer K. (1967). Studies concerning the importance of mononuclear cells in the development of experimental hepatitis. J. Immunol..

[40] Kuriki J., Murakami H., Kakumu S., Sakamoto N., Yokochi T., Nakashima I., Kato N. (1983). Experimental autoimmune hepatitis in mice after immunization with syngeneic liver proteins together with the polysaccharide of *Klebsiella pneumoniae*. Gastroenterology.

[41] Mori Y., Mori T., Yoshida H., Ueda S., Iesato K., Wakashin Y., Wakashin M., Okuda K. (1984). Study of cellular immunity in experimental autoimmune hepatitis in mice. Clin. Exp. Immunol..

[42] Watanabe Y., Kawakami H., Kawamoto H., Ikemoto Y., Masuda K., Takezaki E., Nakanishi T., Kajiyama G., Takeno H. (1987). Effect of neonatal thymectomy on experimental autoimmune hepatitis in mice. Clin. Exp. Immunol..

[43] Lohse A.W., Manns M., Dienes H.P., Meyer zum Buschenfelde K.H., Cohen I.R. (1990). Experimental autoimmune hepatitis: Disease induction, time course and T-cell reactivity. Hepatology.

[44] Toyonaga T., Hino O., Sugai S., Wakasugi S., Abe K., Shichiri M., Yamamura K. (1994). Chronic active hepatitis in transgenic mice expressing interferon-γ in the liver. Proc. Natl. Acad. Sci. USA.

[45] Tiegs G. (1997). Experimental hepatitis and role of cytokines. Acta Gastroenterol. Belg..

[46] Tiegs G., Hentschel J., Wendel A. (1992). A T cell-dependent experimental liver injury in mice inducible by concanavalin A. J. Clin. Investig..

[47] Kusters S., Gantner F., Kunstle G., Tiegs G. (1996). Interferon γ plays a critical role in T cell-dependent liver injury in mice initiated by concanavalin A. Gastroenterology.

[48] Gantner F., Leist M., Lohse A.W., Germann P.G., Tiegs G. (1995). Concanavalin A-induced T-cell-mediated hepatic injury in mice: The role of tumor necrosis factor. Hepatology.

[49] Gorham J.D., Lin J.T., Sung J.L., Rudner L.A., French M.A. (2001). Genetic regulation of autoimmune disease: BALB/c background TGF-β 1-deficient mice develop necroinflammatory IFN- γ-dependent hepatitis. J. Immunol..

[50] Jones-Youngblood S.L., Wieties K., Forman J., Hammer R.E. (1990). Effect of the expression of a hepatocyte-specific MHC molecule in transgenic mice on T cell tolerance. J. Immunol..

[51] Morahan G., Brennan F.E., Bhathal P.S., Allison J., Cox K.O., Miller J.F. (1989). Expression in transgenic mice of class I histocompatibility antigens controlled by the metallothionein promoter. Proc. Natl. Acad. Sci. USA.

[52] Moriyama T., Guilhot S., Klopchin K., Moss B., Pinkert C.A., Palmiter R.D., Brinster R.L., Kanagawa O., Chisari F.V. (1990). Immunobiology and pathogenesis of hepatocellular injury in hepatitis B virus transgenic mice. Science.

[53] Chisari F.V., Ferrari C. (1995). Hepatitis B virus immunopathogenesis. Annu. Rev. Immunol..

[54] Chisari F.V. (1996). Hepatitis B virus transgenic mice: Models of viral immunobiology and pathogenesis. Curr. Top. Microbiol. Immunol..

[55] Christen U., Holdener M., Hintermann E. (2010). Cytochrome P450 2D6 as a model antigen. Dig. Dis..

[56] Zierden M., Kuhnen E., Odenthal M., Dienes H.P. (2010). Effects and regulation of autoreactive CD8^+^ T cells in a transgenic mouse model of autoimmune hepatitis. Gastroenterology.

[57] Ferber I., Schonrich G., Schenkel J., Mellor A.L., Hammerling G.J., Arnold B. (1994). Levels of peripheral T cell tolerance induced by different doses of tolerogen. Science.

[58] Djilali-Saiah I., Lapierre P., Vittozi S., Alvarez F. (2002). DNA vaccination breaks tolerance for a neo-self antigen in liver: A transgenic murine model of autoimmune hepatitis. J. Immunol..

[59] Lapierre P., Djilali-Saiah I., Vitozzi S., Alvarez F. (2004). A murine model of type 2 autoimmune hepatitis: Xenoimmunization with human antigens. Hepatology.

[60] Marceau G., Yang R., Lapierre P., Beland K., Alvarez F. (2014). Low-dose anti-CD3 antibody induces remission of active autoimmune hepatitis in xenoimmunized mice. Liver Int..

[61] Lapierre P., Beland K., Yang R., Alvarez F. (2013). Adoptive transfer of* ex vivo* expanded regulatory T cells in an autoimmune hepatitis murine model restores peripheral tolerance. Hepatology.

[62] Holdener M., Hintermann E., Bayer M., Rhode A., Rodrigo E., Hintereder G., Johnson E.F., Gonzalez F.J., Pfeilschifter J., Manns M.P. (2008). Breaking tolerance to the natural human liver autoantigen cytochrome P450 2D6 by virus infection. J. Exp. Med..

[63] Hardtke-Wolenski M., Fischer K., Noyan F., Schlue J., Falk C.S., Stahlhut M., Woller N., Kuehnel F., Taubert R., Manns M.P. (2013). Genetic predisposition and environmental danger signals initiate chronic autoimmune hepatitis driven by CD4^+^ T cells. Hepatology.

[64] Peters M.G. (2002). Animal models of autoimmune liver disease. Immunol. Cell Biol..

[65] Irla M., Hollander G., Reith W. (2010). Control of central self-tolerance induction by autoreactive CD4^+^ thymocytes. Trends Immunol..

[66] Kobayashi T., Walsh P.T., Walsh M.C., Speirs K.M., Chiffoleau E., King C.G., Hancock W.W., Caamano J.H., Hunter C.A., Scott P. (2003). TRAF6 is a critical factor for dendritic cell maturation and development. Immunity.

[67] Gordon J., Xiao S., Hughes B., Su D.M., Navarre S.P., Condie B.G., Manley N.R. (2007). Specific expression of lacZ and cre recombinase in fetal thymic epithelial cells by multiplex gene targeting at the Foxn1 locus. BMC Dev. Biol..

[68] Corbeaux T., Hess I., Swann J.B., Kanzler B., Haas-Assenbaum A., Boehm T. (2010). Thymopoiesis in mice depends on a Foxn1-positive thymic epithelial cell lineage. Proc. Natl. Acad. Sci. USA.

[69] Aschenbrenner K., D’Cruz L.M., Vollmann E.H., Hinterberger M., Emmerich J., Swee L.K., Rolink A., Klein L. (2007). Selection of Foxp3^+^ regulatory T cells specific for self antigen expressed and presented by Aire^+^ medullary thymic epithelial cells. Nat. Immunol..

[70] Apostolou I., Sarukhan A., Klein L., von Boehmer H. (2002). Origin of regulatory T cells with known specificity for antigen. Nat. Immunol..

[71] Jordan M.S., Boesteanu A., Reed A.J., Petrone A.L., Holenbeck A.E., Lerman M.A., Naji A., Caton A.J. (2001). Thymic selection of CD4^+^CD25^+^ regulatory T cells induced by an agonist self-peptide. Nat. Immunol..

[72] Mathis D., Benoist C. (2009). Aire. Annu. Rev. Immunol..

[73] Akirav E.M., Ruddle N.H., Herold K.C. (2011). The role of AIRE in human autoimmune disease. Nat. Rev. Endocrinol..

[74] Lankisch T.O., Jaeckel E., Strassburg C.P. (2009). The autoimmune polyendocrinopathy-candidiasis-ectodermal dystrophy or autoimmune polyglandular syndrome type 1. Semin. Liver Dis..

[75] Strassburg C.P. (2010). Autoimmune hepatitis. Best Pract. Res. Clin. Gastroenterol..

[76] Clemente M.G., Meloni A., Obermayer-Straub P., Frau F., Manns M.P., de Virgiliis S. (1998). Two cytochromes P450 are major hepatocellular autoantigens in autoimmune polyglandular syndrome type 1. Gastroenterology.

[77] Clemente M.G., Obermayer-Straub P., Meloni A., Strassburg C.P., Arangino V., Tukey R.H., de Virgiliis S., Manns M.P. (1997). Cytochrome P450 1A2 is a hepatic autoantigen in autoimmune polyglandular syndrome type 1. J. Clin. Endocrinol. Metab..

[78] Obermayer-Straub P., Perheentupa J., Braun S., Kayser A., Barut A., Loges S., Harms A., Dalekos G., Strassburg C.P., Manns M.P. (2001). Hepatic autoantigens in patients with autoimmune polyendocrinopathy-candidiasis-ectodermal dystrophy. Gastroenterology.

[79] Anderson M.S., Venanzi E.S., Klein L., Chen Z., Berzins S.P., Turley S.J., von Boehmer H., Bronson R., Dierich A., Benoist C. (2002). Projection of an immunological self shadow within the thymus by the aire protein. Science.

[80] Longhi M.S., Ma Y., Mieli-Vergani G., Vergani D. (2010). Aetiopathogenesis of autoimmune hepatitis. J. Autoimmun..

[81] Czaja A.J. (2010). Autoantibodies as prognostic markers in autoimmune liver disease. Dig. Dis. Sci..

[82] Peiseler M., Sebode M., Franke B., Wortmann F., Schwinge D., Quaas A., Baron U., Olek S., Wiegard C., Lohse A.W. (2012). FOXP3^+^ regulatory T cells in autoimmune hepatitis are fully functional and not reduced in frequency. J. Hepatol..

[83] Taubert R., Hardtke-Wolenski M., Noyan F., Wilms A., Baumann A.K., Schlue J., Olek S., Falk C.S., Manns M.P., Jaeckel E. (2014). Intrahepatic regulatory T cells in autoimmune hepatitis are associated with treatment response and depleted with current therapies. J. Hepatol..

[84] Homberg J.C., Abuaf N., Bernard O., Islam S., Alvarez F., Khalil S.H., Poupon R., Darnis F., Levy V.G., Grippon P. (1987). Chronic active hepatitis associated with antiliver/kidney microsome antibody type 1: A second type of “autoimmune” hepatitis. Hepatology.

[85] Martini E., Abuaf N., Cavalli F., Durand V., Johanet C., Homberg J.C. (1988). Antibody to liver cytosol (anti-LC1) in patients with autoimmune chronic active hepatitis type 2. Hepatology.

[86] Kanzler S., Weidemann C., Gerken G., Lohr H.F., Galle P.R., Meyer zum Buschenfelde K.H., Lohse A.W. (1999). Clinical significance of autoantibodies to soluble liver antigen in autoimmune hepatitis. J. Hepatol..

[87] Hardtke-Wolenski M., Taubert R., Noyan F., Sievers M., Dywicki J., Schlue J., Falk C.S., Lundgren B.A., Scott H.S., Pich A. (2014). Autoimmune hepatitis in a murine APS-1 model is directed against multiple autoantigens. Hepatology.

[88] Anderson M.S., Venanzi E.S., Chen Z., Berzins S.P., Benoist C., Mathis D. (2005). The cellular mechanism of Aire control of T cell tolerance. Immunity.

[89] Senaldi G., Portmann B., Mowat A.P., Mieli-Vergani G., Vergani D. (1992). Immunohistochemical features of the portal tract mononuclear cell infiltrate in chronic aggressive hepatitis. Arch. Dis. Child..

[90] Donaldson P.T. (2004). Genetics of autoimmune and viral liver diseases: Understanding the issues. J. Hepatol..

[91] Parel Y., Chizzolini C. (2004). CD4^+^CD8^+^ double positive (DP) T cells in health and disease. Autoimmun. Rev..

[92] Parel Y., Aurrand-Lions M., Scheja A., Dayer J.M., Roosnek E., Chizzolini C. (2007). Presence of CD4^+^CD8^+^ double-positive T cells with very high interleukin-4 production potential in lesional skin of patients with systemic sclerosis. Arthritis Rheumatol..

[93] Liberal R., Longhi M.S., Grant C.R., Mieli-Vergani G., Vergani D. (2012). Autoimmune hepatitis after liver transplantation. Clin. Gastroenterol. Hepatol..

[94] Crispe I.N. (2011). Liver antigen-presenting cells. J. Hepatol..

[95] Ebrahimkhani M.R., Mohar I., Crispe I.N. (2011). Cross-presentation of antigen by diverse subsets of murine liver cells. Hepatology.

[96] Zhao L., Tang Y., You Z., Wang Q., Liang S., Han X., Qiu D., Wei J., Liu Y., Shen L. (2011). Interleukin-17 contributes to the pathogenesis of autoimmune hepatitis through inducing hepatic interleukin-6 expression. PLoS One.

[97] Yan S., Wang L., Liu N., Wang Y., Chu Y. (2012). Critical role of interleukin-17/interleukin-17 receptor axis in mediating Con A-induced hepatitis. Immunol. Cell Biol..

[98] Lafdil F., Wang H., Park O., Zhang W., Moritoki Y., Yin S., Fu X.Y., Gershwin M.E., Lian Z.X., Gao B. (2009). Myeloid STAT3 inhibits T cell-mediated hepatitis by regulating T helper 1 cytokine and interleukin-17 production. Gastroenterology.

[99] Zenewicz L.A., Yancopoulos G.D., Valenzuela D.M., Murphy A.J., Karow M., Flavell R.A. (2007). Interleukin-22 but not interleukin-17 provides protection to hepatocytes during acute liver inflammation. Immunity.

[100] Crispe I.N. (2012). IL-17 in liver injury: An inflammatory issue?. Immunol. Cell Biol..

[101] Sebode M., Lohse A.W. (2014). Future perspective: Immunomodulatory therapy for autoimmune hepatitis. Dig. Dis..

[102] Oo Y.H., Adams D.H. (2014). Regulatory T cells and autoimmune hepatitis: What happens in the liver stays in the liver. J. Hepatol..

[103] Liberal R., Grant C.R., Holder B.S., Ma Y., Mieli-Vergani G., Vergani D., Longhi M.S. (2012). The impaired immune regulation of autoimmune hepatitis is linked to a defective galectin-9/tim-3 pathway. Hepatology.

[104] Knolle P.A., Schmitt E., Jin S., Germann T., Duchmann R., Hegenbarth S., Gerken G., Lohse A.W. (1999). Induction of cytokine production in naive CD4^+^ T cells by antigen-presenting murine liver sinusoidal endothelial cells but failure to induce differentiation toward Th1 cells. Gastroenterology.

[105] Lohse A.W., Knolle P.A., Bilo K., Uhrig A., Waldmann C., Ibe M., Schmitt E., Gerken G., Meyer Zum Buschenfelde K.H. (1996). Antigen-presenting function and B7 expression of murine sinusoidal endothelial cells and Kupffer cells. Gastroenterology.

[106] Carambia A., Frenzel C., Bruns O.T., Schwinge D., Reimer R., Hohenberg H., Huber S., Tiegs G., Schramm C., Lohse A.W. (2013). Inhibition of inflammatory CD4 T cell activity by murine liver sinusoidal endothelial cells. J. Hepatol..

[107] Kruse N., Neumann K., Schrage A., Derkow K., Schott E., Erben U., Kuhl A., Loddenkemper C., Zeitz M., Hamann A. (2009). Priming of CD4^+^ T cells by liver sinusoidal endothelial cells induces CD25^low^ forkhead box protein 3^−^ regulatory T cells suppressing autoimmune hepatitis. Hepatology.

